# Rabi oscillations in a stretching molecule

**DOI:** 10.1038/s41377-023-01075-9

**Published:** 2023-02-02

**Authors:** Shengzhe Pan, Chenxi Hu, Wenbin Zhang, Zhaohan Zhang, Lianrong Zhou, Chenxu Lu, Peifen Lu, Hongcheng Ni, Jian Wu, Feng He

**Affiliations:** 1 State Key Laboratory of Precision Spectroscopy, East China Normal University, Shanghai, 200241 China; 2 Key Laboratory for Laser Plasmas (Ministry of Education) and School of Physics and Astronomy, Collaborative Innovation Center of IFSA (CICIFSA), Shanghai Jiao Tong University, Shanghai, 200240 China; 3 Collaborative Innovation Center of Extreme Optics, Shanxi University, Taiyuan, Shanxi 030006 China; 4 Chongqing Key Laboratory of Precision Optics, Chongqing Institute of East China Normal University, Chongqing, 401121 China; 5 CAS Center for Excellence in Ultra-intense Laser Science, Shanghai, 201800 China

**Keywords:** Ultrafast photonics, Nonlinear optics

## Abstract

Rabi oscillation is an elementary laser-driven physical process in atoms and artificial atoms from solid-state systems, while it is rarely demonstrated in molecules. Here, we investigate the bond-length-dependent Rabi oscillations with varying Rabi frequencies in strong-laser-field dissociation of H_2_^+^. The coupling of the bond stretching and Rabi oscillations makes the nuclei gain different kinetic energies while the electron is alternatively absorbing and emitting photons. The resulting proton kinetic energy spectra show rich structures beyond the prediction of the Floquet theorem and the well-accepted resonant one-photon dissociation pathway. Our study shows that the laser-driven Rabi oscillations accompanied by nuclear motions are essential to understanding the bond-breaking mechanism and provide a time-resolved perspective to manipulate rich dynamics of the strong-laser-field dissociation of molecules.

## Introduction

Over eighty years ago, Rabi oscillations were proposed to describe the strong coupling and population transfer in a two-level quantum system exposed to an oscillatory driving field^1^. From then on, Rabi oscillation plays an essential role in abundant physical phenomena and is considered as one of the most fundamental processes in light-matter interactions with a characterized frequency given by the laser parameters as well as the energy gap and dipole between two involved electronic states. It is utilized to control the quantum state in a single quantum dot^2–4^, lay the foundation for quantum computation^5^, realize the coherent phase modulation of free-electron states^6^, observe the many-atom entangled state in an optical-clock transition^7^, and enhance the collective atom-light coupling^8,9^. With the advent of intense ultrafast laser pulses, one is able to observe the Rabi oscillation of resonant state in continuum spectra^10,11^, also named as Autler-Townes splitting^12^. Recently, by utilizing the Freeman resonance^13^, the femtosecond two-photon Rabi oscillations in atoms have been demonstrated^14^. The laser-driven transient dipole response was experimentally reconstructed^15^, and the breaking of the area theorem of the carrier-wave Rabi flopping^16^ was observed in the high-harmonic generation of atoms^17^.

As compared to atoms, molecules have an extra degree of vibration, which adds an additional knob to the Rabi oscillations in light-molecule interactions. Taking H_2_^+^ as an example, during its stretching or dissociation, the energy gap and the dipole between the ground (1*sσ*_*g*_) and excited (2*pσ*_*u*_) states vary, leading to the bond-length-dependent Rabi frequency for a given laser field. Though Rabi oscillations in atoms and solid-state systems have been extensively studied, surprisingly, only a few theoretical and experimental works reported Rabi oscillations in molecules. In 1996, the two-photon Rabi oscillation in a three-level molecular system was demonstrated in both theory and experiment^18^. In 1999, the Rabi oscillation among dissociative states of a Na_2_^+^ molecule was theoretically explored^19^ but yet lacked experimental observations yet. It is recently predicted that the laser-driven Rabi oscillation in H_2_^+^ depends on the molecular orientation, and thus the dissociative fragments present angular nodes in their angular distributions^20^. Generally, Rabi oscillations in a molecule inevitably couple with the nuclear motion^21^. However, how such a coupling determines the kinetic energy release (KER) in the dissociative fragments is still an open question.

**Fig. 1:**
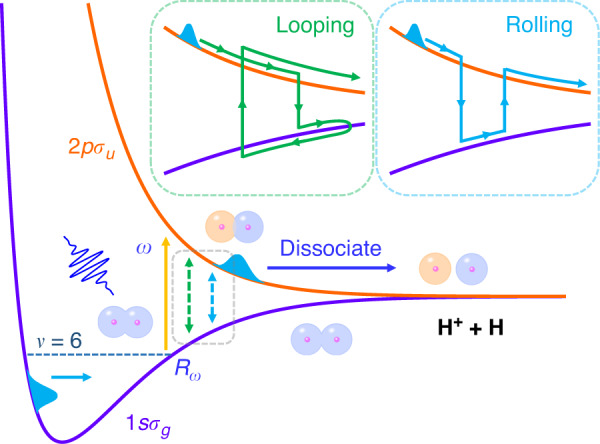
Schematic illustration of dynamical Rabi coupling in strong-field dissociation of H_2_^+^. Violet and orange curves denote the potential energy curves of the electronic ground (1*sσ*_*g*_) and excited (2*pσ*_*u*_) states, respectively. The yellow vertical arrow between the two curves indicates the resonant one-photon transition at *R*_*ω*_. The two insets sketch the looping and rolling processes during the molecular dissociation, marked by the gray frame. The dashed horizontal line marks the eigenenergy of the *υ* = 6 vibrational state on the 1*sσ*_*g*_ potential energy curve

Here we unveil the importance of the dynamical Rabi coupling in strong-laser-field dissociation of H_2_^+^ and explore intriguing dissociation dynamics beyond the well-accepted resonant one-photon dissociation scenario^22–35^. During the dissociation, coupled with the laser field, the electron hops between the 1*sσ*_*g*_ and 2*pσ*_*u*_ states, forming the Rabi oscillations. Meanwhile, as illustrated in Fig. 1, the nuclear wave packet (NWP) may propagate alternatively along the two potential energy curves towards a larger internuclear distance monotonically, termed as the rolling process, or may propagate outwards along the 2*pσ*_*u*_ potential energy curve followed by the inward propagation in the 1*sσ*_*g*_ potential curve and then be relaunched to 2*pσ*_*u*_ state again followed by subsequent dissociation, termed as the looping process. The rolling and looping dissociation pathways lead to different KERs of the ejected protons. In the following, we first verify the role of the Rabi oscillations in strong-laser-field dissociation of H_2_^+^ and then analyze the proton energy spectra determined by the rolling and looping processes, which are afterward compared with the experimental measurements.

## Results

### Verifying laser-driven Rabi oscillations in molecular dissociation

**Fig. 2:**
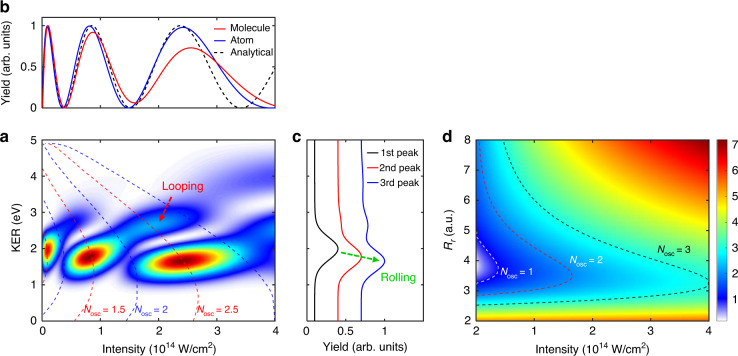
Observing dynamical Rabi coupling using a few-cycle laser pulse. **a** The simulated KER spectra of dissociative fragments as a function of the peak intensity of a four-optical-cycle 400-nm laser pulse. Red and blue dashed curves denote the predicted KER using Eq. (3), where the molecule undergoes half-integral and integral Rabi oscillations at different internuclear distances, respectively. **b** Populations of the 2*pσ*_*u*_ state after the laser-H_2_^+^ interaction, i.e., the proton yield, as a function of the laser intensity. The red curve is obtained by integrating the two-dimensional map in **a** over the region of 1.5 eV < KER < 2.5 eV. The blue curve is numerically simulated by only switching on the transition at *R*_*ω*_. The black dashed curve is analytically calculated using Eq. (1). The maxima of all the curves are normalized to one for the convenience of comparison. **c** The dissociation yields as a function of the KER obtained by integrating the two-dimensional map in (**a**) within the laser intensity regions of [0, 3.7 × 10^13^], [3.7 × 10^13^, 1.5 × 10^14^], and [1.5 × 10^14^, 4 × 10^14^] W/cm^2^ corresponding to the three peaks. **d** Number of Rabi oscillations *N*_osc_ as a function of the laser intensity and internuclear distance. White, red, and black dashed curves denote the positions where *N*_osc_ = 1, 2, and 3, respectively. The initial state in the calculation is the *v* = 6 vibrational state

The dissociation of H_2_^+^ is governed by the laser coupling of the two lowest electronic states. We, therefore, simulate the two-level time-dependent Schrödinger equation (TDSE) (see “Methods”) and trace the wave function evolution to unveil the mechanism. We start from the *v* = 6 vibrational state on the 1*sσ*_*g*_ potential energy curve since such an NWP has the maximum population at around *R*_*ω*_ = 3.8 to embrace the well-accepted one-photon resonant excitation^26,33,34,36–38^, as illustrated in Fig. 1. The laser pulse has four optical cycles and the central wavelength is 400 nm. Figure 2a shows the laser-intensity-dependent proton KER spectrum of the laser-dissociated H_2_^+^. The vertical integration of Fig. 2a over the region of 1.5 eV < KER < 2.5 eV gives the intensity-dependent dissociation probability, as shown by the solid red curve in Fig. 2b. In Fig. 2a, the concentrated yield of protons around 2.0 eV in the KER spectrum at weak laser intensities originates from the dissociation via the one-photon resonant transition between 1*sσ*_*g*_ and 2*pσ*_*u*_ states at around *R*_*ω*_ = 3.8^26,33,34,36–38^, termed as the resonant one-photon dissociation pathway, which is however completely suppressed at the laser intensity of 4 × 10^13^ W/cm^2^. In both Figs. 2a and 2b, one can see that the dissociation probability oscillates with the increasing laser intensity. To understand such a strong modulation of the intensity-dependent dissociation yield, we perform TDSE simulations by temporarily freezing the nuclear motion and fixing the internuclear distance to be *R*_*ω*_, i.e., only switching on the dipole transition at *R*_*ω*_. Such a treatment is analogous to perform a simulation in atomic systems. The atomic-like calculations give the population on the 2*pσ*_*u*_ state at the end of the laser pulse, as shown by the blue curve in Fig. 2b.

In the atomic-like simulation, within the rotating wave approximation omitting the high-frequency term and reserving the near-resonance low-frequency term^39^, one can analytically write down the time-dependent population of the 2*pσ*_*u*_ state at a given internuclear distance *R*_*r*_ where Rabi oscillations occur^39^ as follows

1 $$P_u\left( {I_0,R_r,t} \right) = \frac{{E_0^2D^2\left( {R_r} \right)}}{{\omega _r^2\left( {I_0,R_r} \right)}}\sin ^2\left[ {\frac{{\omega _r\left( {I_0,R_r} \right)}}{2}t} \right]$$

where $\omega _r\left( {I_0,R_r} \right) = \sqrt {\left[ {E_0D\left( {R_r} \right)} \right]^2 + \left[ {V_u\left( {R_r} \right) - V_g\left( {R_r} \right) - \omega } \right]^2}$ denotes the Rabi frequency as a function of the laser peak intensity *I*_0_ = *E*_0_^2^ and the internuclear distance *R*_*r*_ considering the nuclear motion of H_2_^+^. According to Eq. (1), the population of the 2*pσ*_*u*_ state approaches maximum when $\frac{{\omega _r\left( {I_0,R_r} \right)}}{2}t = \left( {n + \frac{1}{2}} \right)\pi$, with *n* an integer starting from 0. Thus, the number of Rabi oscillations at the end of the laser pulse is described as

2 $$N_{{{{\mathrm{osc}}}}}\left( {I_0,R_r} \right) = \frac{{\omega _r\left( {I_0,R_r} \right)\tau _{{{{\mathrm{eff}}}}}}}{{2\pi }} = \frac{{\omega _r\left( {I_0,R_r} \right)N_{{{{\mathrm{eff}}}}}}}{\omega }$$

where *τ*_eff_ = *N*_eff_*T* denotes the effective duration of the laser pulse for a square-of-sine-shaped envelope, which means that only the central part of the laser pulse is strong enough to induce the Rabi oscillation. Here, by setting the effective optical cycle of *N*_eff_ = 1.88, the population of the 2*pσ*_*u*_ state governed by Eq. (1) as a function of the laser intensity, shown as the black curve, agrees with the other two curves in Fig. 2b. All these agreements verify the Rabi oscillation in the dissociation of H_2_^+^. Integral (half-integral) Rabi oscillations lead to the minimum (maximum) population of the 2*pσ*_*u*_ state and thus determines the yield of the protons around 2.0 eV.

### KER spectra determined by rolling and looping dynamics

After verifying the Rabi oscillations in strong-laser-field dissociation of H_2_^+^, we now analyze how it alters the KER of the ejected protons. Compared to atoms, Rabi frequency in molecular dissociation depends on the bond length. More importantly, the bond stretching and Rabi oscillations are inevitably coupled, which challenges our understanding of the KER spectrum based on the well-accepted dissociation scenario. In Fig. 2a, in addition to the main peak located around 2.0 eV, one may see the high-energy tails above 2.5 eV guided by the red arrow and the low-energy shifts below 2.0 eV guided by the green arrow in Fig. 2c. The KER spectra labeled with different peaks in Fig. 2c are obtained from Fig. 2a by horizontally integrating over different intensity regions, i.e., [0, 3.7 × 10^13^], [3.7 × 10^13^, 1.5 × 10^14^] and [1.5 × 10^14^, 4 × 10^14^] W/cm^2^. Such unexpected characters can be understood by tracing the Rabi oscillations in the time domain. As illustrated in the insets in Fig. 1, once the H_2_^+^ is pumped to the 2*pσ*_*u*_ state from the 1*sσ*_*g*_ state, the NWP may move outwards and gain kinetic energy. Besides the direct dissociation along the 2*pσ*_*u*_ potential energy curve, this NWP may transit back to the 1*sσ*_*g*_ state at a large internuclear distance due to the Rabi oscillation. Then, the NWP continuously moves outwards along the 1*sσ*_*g*_ curve, followed either by being pumped again to the 2*pσ*_*u*_ state at a larger internuclear distance, or by decelerating and then reversing its moving direction before being transited again to the 2*pσ*_*u*_ state at a smaller internuclear distance followed by subsequent dissociation. These two scenarios are named as rolling and looping processes, respectively. The looping and rolling dissociation pathways end with protons carrying larger and smaller kinetic energies, respectively, compared to the resonant one-photon pathway.

To quantitatively understand the KERs induced by the aforementioned rolling and looping processes illustrated in Fig. 1, we formulate the KER of the dissociating NWP as

3 $${{{\mathrm{KER}}}} = E_v + {{\Delta }}V\left( {R_\omega } \right) - {{\Delta }}V\left( {R_r} \right) + {{\Delta }}V(R_{r^\prime }) - V_u( + \infty )$$

where *E*_*v*_ denotes the eigenenergy of the *v* = 6 vibrational state on the 1*sσ*_*g*_ potential energy curve, *R*_*ω*_, *R*_*r*_ and $R_{r^\prime }$ denote the transition internuclear distances, Δ*V*(*R*) denotes the *R*-dependent energy gap between 1*sσ*_*g*_ and 2*pσ*_*u*_ states, and *V*_*u*_(+∞) denotes the dissociation limit of the 2*pσ*_*u*_ state. Here, the critical internuclear distances *R*_*r*_ and $R_{r^\prime }$ are determined by the nuclear motion at the most probable instant where the population of the 2*pσ*_*u*_ state achieves another maximum after the first one-photon transition from 1*sσ*_*g*_ to 2*pσ*_*u*_ states at *R*_*ω*_. According to Eq. (2), one may retrieve the internuclear distances *R*_*r*_ to accomplish integral or half-integral Rabi oscillations if the laser pulse is given. For example, the critical internuclear distances for integral Rabi oscillations (*N*_osc_ = 1, 2, and 3) are indicated as the dashed curves in Fig. 2d. Based on these critical internuclear distances, we can predict the maxima and minima of the proton yield as a function of the KER and laser intensity. For instance, considering the looping dynamics where the inward-moving NWP on the 1*sσ*_*g*_ state is assumed to undergo another one-photon transition at *R*_*ω*_, Eq. (3) can be written as

4 $${{{\mathrm{KER}}}} = E_v + {{\Delta }}V\left( {R_\omega } \right) - {{\Delta }}V\left( {R_r} \right) + {{\Delta }}V(R_\omega ) - V_u( + \infty )$$

According to the simplified formula Eq. (4), the proton KERs from the dissociation of H_2_^+^ undergoing integral or half-integral Rabi oscillations as a function of the laser intensity are plotted with blue and red dashed curves in Fig. 2a. The red and blue dashed curves precisely predict the location of the maximum and minimum proton yields, respectively. The excellent accordance between the analytical calculation and numerical simulation verifies the origin of the high-energy and low-energy protons, i.e., produced via the looping and rolling pathways ensured by the bond-length-dependent dynamical Rabi coupling.

### Pulse-length-dependent Rabi oscillations

**Fig. 3:**
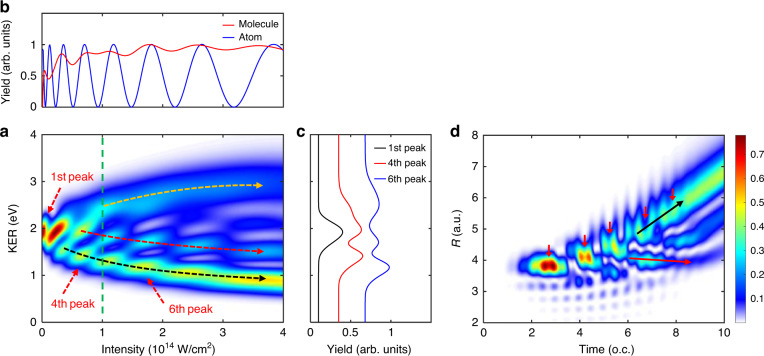
Verifying rolling and looping dynamics in a multicycle laser pulse. **a** The simulated KER spectra as a function of the peak intensity of a ten-optical-cycle 400-nm laser pulse. **b** Populations of the 2*pσ*_*u*_ state after the laser-H_2_^+^ interaction, i.e., the proton yield, as a function of the laser intensity. The red curve is obtained by integrating the two-dimensional map in (**a**) over the whole KER region. The blue curve is numerically simulated by only switching on the dipole transition at *R*_*ω*_. The maxima of both curves are normalized to one for the convenience of comparison. **c** The dissociation yields as a function of the KER obtained by integrating the two-dimensional map in (**a**) within the laser intensity regions of [0, 1 × 10^12^], [5 × 10^13^, 9 × 10^13^] and [1.3 × 10^14^, 2 × 10^14^] W/cm^2^, corresponding to the 1st, 4th and 6th peaks, respectively. **d** Temporal evolution of the density distribution of *χ*_*u*_ calculated with the laser intensity *I*_0_ = 1 × 10^14^ W/cm^2^, labeled by the green dashed line in (**a**). The five small red arrows denote that the outward NWP undergoes five Rabi oscillations. The initial state in the calculation is the *v* = 6 vibration state

Such dynamical Rabi coupling becomes more prominent if the driving laser pulse has a longer duration since more Rabi oscillations will be involved during the stretching of the molecule. Figure 3a shows the intensity-dependent proton KER spectrum driven by a ten-optical-cycle laser pulse with a central wavelength of 400 nm. The proton yield integrated over the whole energy region as a function of laser intensity is plotted by the red curve in Fig. 3b. Note that the red curve has been normalized by its maximum. The corresponding atomic-like calculation is shown by the blue curve in Fig. 3b with the same laser parameters. The smaller modulation depth of the red curve here compared to the red one in Fig. 2b is undoubtedly attributed to the more distinct nuclear motion in a longer laser pulse. Multiple Rabi oscillations bring several looping processes, which may mix with the rolling process, resulting in complex KER distributions. The low KER distribution, guided by the black arrow in Fig. 3a, shows a clear tendency that the energy peaks shift lower monotonically with the increasing laser intensity induced by the pure rolling pathway. On the contrary, the branch having the highest KER, as guided by the yellow arrow in Fig. 3a, is due to the pure looping process. The mixture of looping and rolling processes contributes to the KER spectra between these two branches. The most prominent mixed pathway is the first-order looping pathway, meaning that there is only one looping process happening during the whole rolling process, guided by the red arrow in Fig. 3a. It should be mentioned that the mixed pathway is a sequential process of looping and rolling. However, the order of these two processes may depend on the instantaneous field strength and nuclear momentum when the NWP reaches critical internuclear distances for Rabi oscillations.

As indicated in Fig. 1, the NWP must move inward in the looping process, and we now trace the propagation details of the NWP during the dissociation. In Fig. 3d, we show the wave packet propagation on the 2*pσ*_*u*_ potential energy curve driven by the ten-optical-cycle laser pulse with a peak intensity of 1 × 10^14^ W/cm^2^. The main trace of the NWP undergoes five Rabi oscillations, marked by the five small red arrows in Fig. 3d, contributing to the fifth rolling peak, guided by the vertical green dashed line in Fig. 3a, at *I*_0_ = 1 × 10^14^ W/cm^2^. Looking into the details in Fig. 3d more closely, one may observe two sub-traces denoting one pathway of moving outwards and the other of moving inwards, guided by black and red arrows, respectively. As already described in Fig. 1, for the looping process, once the NWP moves inwards on the 1*sσ*_*g*_ potential curve, it will continuously move inward even after it is relaunched to the 2*pσ*_*u*_ state until it is decelerated to rest. Such a looping process is guided by the red arrow in Fig. 3d. Generally, more intense lasers induce more Rabi oscillations. Thus, the NWP experiences more propagation along the 1*sσ*_*g*_ curve to conquer the dissociation limit, resulting in smaller kinetic energy release in the rolling process. On the contrary, in the looping processes, once the NWP propagates inwards on the 1*sσ*_*g*_ curve, it will acquire more kinetic energies after being relaunched to the 2*pσ*_*u*_ curve at a smaller internuclear distance.

The direct visualization of the dynamical Rabi coupling has been verified using laser fields with another wavelength in TDSE simulations. For any wavelength of the incident laser pulse, the initial vibrational state of H_2_^+^ is selected to meet the condition of the one-photon resonant transition. The energy gap between two electronic states at the internuclear distance where the distribution of the resonant vibrational state is maximum is equal to the one-photon energy, producing the maximum dissociation probability^26^.

### Experimental measurements

**Fig. 4:**
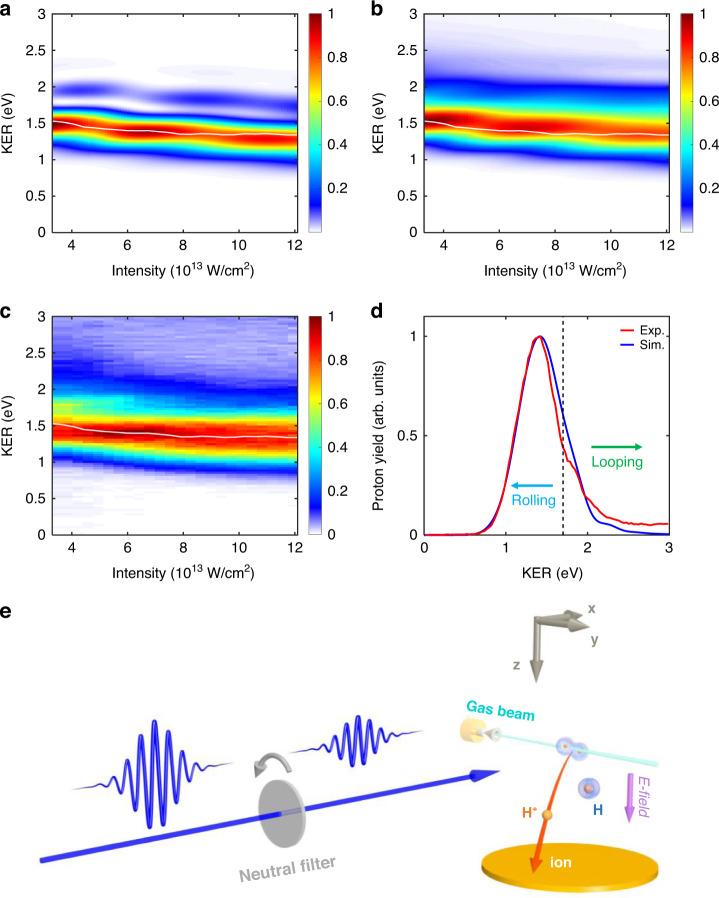
Probing rolling and looping dynamics via comparing experiments with simulations. **a** The simulated KER spectra as a function of the peak intensity of a ten-optical-cycle 400-nm laser pulse. The initial state is the superimposed vibrational state weighted by Frank-Condon factors, which freely propagates 6 fs before the laser pulse acts. **b** Same as (**a**) but averaged over free propagation time of ^2,10^ fs of the initial state. **c** The experimentally measured KER spectra at different laser intensities. The white curves in (**a**–**c**) illustrate the mean values of the KER spectra at different laser intensities. **d** The asymmetric KER spectra obtained by integrating the signals in (**b**) and (**c**) over all laser intensities. The low- and high-energy regions partitioned by the black dashed line are labeled as rolling and looping pathways. The maxima of all the spectra are normalized to one for the convenience of comparison. **e** Schematic illustration of the experimental set-up

Based on the above understanding of the rolling and looping processes, we conceive an experiment using the COLTRIMS technique (See “Methods”) to observe such scenarios. Experimentally, H_2_ is photoionized and the generated H_2_^+^ is afterward dissociated by the incident laser pulse. In order to compare with experimental measurements, in the following numerical simulation, the initial state is set to be the superimposed vibrational states weighted by the Frank-Condon factors, mimicking the NWP just after the single ionization of H_2_. Figure 4a presents the proton KER spectrum in the dissociation of H_2_^+^ driven by a ten-optical-cycle 400-nm laser pulse with different peak intensities ranging from *I*_0_ = 3.3 × 10^13^ W/cm^2^ to 1.2 × 10^14^ W/cm^2^. Here, the superimposed Frank-Condon state firstly freely propagates 6 fs to move outwards on the 1*sσ*_*g*_ potential energy curve, and then the laser pulse is introduced to generate the laser-molecule coupling mainly around the internuclear distances in favor of Rabi oscillations. Hence, the main three-peak structures at *I*_0_ = 4 × 10^13^, 7 × 10^13^, and 1.1 × 10^14^ W/cm^2^ in Fig. 4a are similar to those in Fig. 3a. The aforementioned low-energy and high-energy structures can be distinguished by two clear traces in Fig. 4a, where the low-energy rolling pathway is almost the same as the one in Fig. 3a. While different from the multi-order looping structures with higher KERs in Fig. 3a, only the first-order looping pathway is observed in Fig. 4a, which is mainly ascribed to the nuclear movement. When the NWP weighted by the Frank-Condon factors moves to the internuclear distances for effective Rabi oscillations, the NWP already obtains non-negligible velocity moving outwards. Thus, such an outward velocity makes it hard to reverse its moving direction, and thus the looping pathway is relatively suppressed. Considering that H_2_ can be photoionized in each optical cycle in the whole laser pulse, we freely propagate the NWP for different periods before introducing the laser pulse to dissociate H_2_^+^. Numerically, we run different simulations by freely propagating the NWPs in a range of ^2,10^ fs and average the KER spectrum from the 2*pσ*_*u*_ state, as shown in Fig. 4b. The looping and rolling branches are blurred in Fig. 4b due to the energy overlap in simulations using different free propagation time.

The experimental KER spectra of the dissociative fragments of H_2_^+^ as a function of the laser intensity are shown in Fig. 4c. The white curve in Fig. 4c is the mean value of the KER spectrum as a function of the laser intensity, which is also plotted in Fig. 4a, b for comparison. The experimental results demonstrate a similar structure as the simulation results, especially the aforementioned mean-KER trace and the asymmetric KER spectra in Fig. 4d, which are horizontally integrated over the laser intensities from Fig. 4b, c. The green and blue arrows in Fig. 4d are used to divide the contribution of the nuclear looping and rolling dynamics in the experimental KER spectrum, respectively. Although the periodical oscillation is blurred in experiments due to the averaging effect, the monotonic decrease of KER with the increasing laser intensity agrees with the simulation results, verifying the looping and rolling scenarios, and hence supporting the Rabi oscillation in molecular systems.

## Discussion

The Rabi oscillations in molecular dissociation demand a laser intensity between the perturbative and ionization regime. Only the one-photon dissociation pathway is noticeable in the perturbative regime, where the laser intensity is smaller than 1 × 10^13^ W/cm^2^. The perturbative theory is good enough to describe the coupling between the 1*sσ*_*g*_ and 2*pσ*_*u*_ states^26^. While in the ionization regime, where the laser intensity is higher than 3 × 10^14^ W/cm^2^, the laser field can ionize the majority of the H_2_^+^ molecules. Thus the ionization dynamics will influence the dissociation dynamics, and the two-level model needs to be amended. For the laser parameters we used in the theory-experiment joint study, though the peak intensity is up-limited to about 1.3 × 10^14^ W/cm^2^, the dissociation happens at the falling edge of the pulse^40^, where the field-induced ionization can be neglected.

In many fields where Rabi oscillations have been fully investigated, such as quantum optics and quantum dots, the external laser field is weak and has a long-time duration to maintain the high energy resolution and finely control the energy level. While in the ultrafast and strong-field community, a strong femtosecond laser pulse is often utilized to induce the Rabi oscillations and even to observe the ultrafast dynamics of atoms and molecules in femtosecond time scales at the cost of the energy resolution due to the time-energy uncertainty.

In retrospect, our explanation based on the coupling of nuclear movement and electron Rabi oscillations is fundamentally different from the conventional one-photon bond-softening scenario. Using the Floquet formalism^41,42^, different dissociation pathways have been well recognized^22,43–46^, and it is suggested that lower vibrational states can spill out the potential energy curves when a driving laser pulse becomes stronger. The Floquet formalism works well when the driving laser is a continuum wave, which unambiguously identifies the dissociation pathways from the energy point of view by paying for the loss of time information. On the contrary, we propose a fundamentally different scenario upon Rabi oscillations to explore more fruitful dynamical processes beyond the previous studies. In the Rabi oscillation scenario, H_2_^+^ may first absorb one photon and then transit from the 1*sσ*_*g*_ state to the 2*pσ*_*u*_ state. Once the accumulated population in the 2*pσ*_*u*_ state is larger than that in the 1*sσ*_*g*_ state, H_2_^+^ in the 2*pσ*_*u*_ state may emit one photon and dump to the 1*sσ*_*g*_ state, forming a complete Rabi oscillation. During the dissociation, if the NWP undergoes half-integer multiples of Rabi oscillations, the proton will end with the net-one-photon absorption, which is similar to the bond-softening scenario only from the energy point of view. However, if integer multiples of the Rabi oscillation have been accomplished, H_2_^+^ dissociates along the 1*sσ*_*g*_ curve in accordance with the net-zero-photon dissociation pathway^37,47,48^.

In the dissociation of H_2_^+^, the laser-driven Rabi oscillations between the two lowest electronic states during the stretching of the molecular bond alter the well-known one-photon resonant dissociation pathway and enrich the KER spectrum of dissociative fragments. Based on the analysis of the propagation of the NWP on the potential energy curves, the nuclear looping and rolling dynamics ensured by the dynamical Rabi coupling of the two lowest electronic states in a stretching H_2_^+^ are clearly identified in the TDSE simulations and further verified in experiments. The dynamical Rabi coupling is an essential step of the well-known Rabi oscillations from atoms to molecules, giving birth to a complete understanding of the laser-driven molecular dissociation, particularly the ejection of slow nuclear fragments. It also provides a time-resolved perspective to understand the ultrafast processes in molecular dissociation beyond the Floquet theorem. The electron hopping mechanism presented here is general for strong-field dynamics of a stretching molecule, which also has implications for complex molecular processes, including the nuclear-electron correlations.

## Materials and methods

### Quantum simulations

For H_2_^+^, the 1*sσ*_*g*_ and 2*pσ*_*u*_ potential energy curves are far from others, and these two states generally govern the dissociation. Theoretically, we study its strong-laser-field dissociation by simulating the two-level one-dimensional time-dependent Schrödinger equation (TDSE)^49^ with the Born-Oppenheimer approximation (atomic units are used throughout unless stated otherwise):

5 $$i\frac{\partial }{{\partial t}}\left( {\begin{array}{*{20}{c}} {\chi _g\left( {R,t} \right)} \\ {\chi _u\left( {R,t} \right)} \end{array}} \right) = \left( {\begin{array}{*{20}{c}} {\hat T + V_g\left( R \right)} & {D(R)E\left( t \right)} \\ {D\left( R \right)E\left( t \right)} & {\hat T + V_u\left( R \right)} \end{array}} \right)\left( {\begin{array}{*{20}{c}} {\chi _g\left( {R,t} \right)} \\ {\chi _u\left( {R,t} \right)} \end{array}} \right)$$

Here the dipole approximation and the length gauge are adopted. *χ*_*g*_(*R*, *t*) and *χ*_*u*_(*R*, *t*) are NWPs associated with the electron in the 1*sσ*_*g*_ and 2*pσ*_*u*_ states, respectively. *V*_*g*_(*R*) and *V*_*u*_(*R*) are the 1*sσ*_*g*_ and 2*pσ*_*u*_ potential energy curves, respectively, as sketched in Fig. 1. *R* is the internuclear distance, $\hat T$ is the nuclear kinetic energy operator, *D*(*R*) is the *R*-dependent transition dipole between the two states, and *E*(*t*) is the electric field of the incident laser pulse. Molecular rotation is neglected in this model. The time and spatial steps are *dt* = 0.1 and *dR* = 0.02, respectively. *R* spans the range [0, 100]. The simulation box is large enough to hold all dissociating NWPs and thus no absorbing boundaries are used. The initial state is obtained using the imaginary-time propagation algorithm^50^, and the Crank-Nicolson method^51^ is adopted to propagate the NWP in real time. The laser pulse is written as *E*(*t*) = *E*_0_sin^2^(*πt*/*τ*)cos(*ωt*), where *E*_0_ is the electric field amplitude, *ω* is the laser frequency, and *τ* is the duration of the laser pulse. The laser polarization direction is parallel to the molecular axis. The initial state will be chosen according to concrete calculations. The dissociation yield is mainly attributed to the population of the 2*pσ*_*u*_ state at the end of the simulation, i.e., $P_u = {\int} {\left| {\chi _u(R,t_{end})} \right|} ^2dR$. The KER spectra are calculated from the momentum distribution, i.e., $\left| {\phi _u\left( {p,t_{end}} \right)} \right|^2/p$, where $\phi _u(p,t_{end})$ denotes the Fourier transform of the position-dependent nuclear wave function. In the two-level simulation, the electron is restricted to the two bound states, and thus the ionization of H_2_^+^ is neglected. We have examined that the ionization rate is minimal compared to the dissociation rate in this theory-experiment joint work using a TDSE simulation with a model including ionization^52^.

### Experimental details

We perform the experiment in an ultrahigh vacuum chamber of a cold target recoil ion momentum spectrometer (COLTRIMS)^53,54^. A linearly polarized ultraviolet laser pulse (46 fs at FWHM, 395 nm) focused into the apparatus is produced by frequency doubling the near-infrared femtosecond laser pulse delivered from a multi-pass Ti:sapphire laser system in a *β*-barium borate crystal. A supersonic gas beam of H_2_ with a driving pressure of 2.0 bar is injected into the vacuum chambers and cooled to tens of Kelvins after the adiabatic expansion through a 30-*µ*m nozzle. The H_2_ target molecule is firstly ionized and subsequently dissociates into a proton (H^+^) and a hydrogen atom (H), where the charged proton can be accelerated with a weak (~14.7 V/cm) static electric field and detected by a time- and position-sensitive microchannel plate detector at the end of the spectrometer, as shown in Fig. 4e. The peak intensity of the laser pulse in the interaction region is adjusted by a neutral filter in the beamline, ranging from *I*_0_ = 3.3 × 10^13^ W/cm^2^ to 1.2 × 10^14^ W/cm^2^. To avoid mixing the dissociation yield of the molecules not oriented along the laser polarization, in the experiment, we select the dissociative fragments along the laser polarization within a small range, i.e., |*ϕ*_ion_ – *ϕ*_*E*_| < 5°, where *ϕ*_ion_ and *ϕ*_*E*_ denote the emission direction of dissociative fragments H^+^ and the polarization direction of the laser pulse, respectively.

## Data Availability

All data are available from the corresponding authors upon reasonable request.

## References

[1] Rabi II (1936). On the process of space quantization. Phys. Rev..

[2] Stievater TH (2001). Rabi oscillations of excitons in single quantum dots. Phys. Rev. Lett..

[3] Yoshie T (2004). Vacuum Rabi splitting with a single quantum dot in a photonic crystal nanocavity. Nature.

[4] Press D (2008). Complete quantum control of a single quantum dot spin using ultrafast optical pulses. Nature.

[5] Blais A (2004). Cavity quantum electrodynamics for superconducting electrical circuits: an architecture for quantum computation. Phys. Rev. A.

[6] Feist A (2015). Quantum coherent optical phase modulation in an ultrafast transmission electron microscope. Nature.

[7] Pedrozo-Peñafiel E (2020). Entanglement on an optical atomic-clock transition. Nature.

[8] Lukin MD (2001). Dipole blockade and quantum information processing in mesoscopic atomic ensembles. Phys. Rev. Lett..

[9] Dudin YO (2012). Observation of coherent many-body Rabi oscillations. Nat. Phys..

[10] Sun ZG, Lou N (2003). Autler-Townes splitting in the multiphoton resonance ionization spectrum of molecules produced by ultrashort laser pulses. Phys. Rev. Lett..

[11] Palacios A, Bachau H, Martín F (2006). Step-ladder Rabi oscillations in molecules exposed to intense ultrashort vuv pulses. Phys. Rev. A.

[12] Autler SH, Townes CH (1955). Stark effect in rapidly varying fields. Phys. Rev..

[13] Freeman RR (1987). Above-threshold ionization with subpicosecond laser pulses. Phys. Rev. Lett..

[14] Fushitani M (2016). Femtosecond two-photon Rabi oscillations in excited He driven by ultrashort intense laser fields. Nat. Photonics.

[15] Stooß V (2018). Real-time reconstruction of the strong-field-driven dipole response. Phys. Rev. Lett..

[16] Hughes S (1998). Breakdown of the area theorem: carrier-wave Rabi flopping of femtosecond optical pulses. Phys. Rev. Lett..

[17] Ciappina MF (2015). Carrier-wave Rabi-flopping signatures in high-order harmonic generation for alkali atoms. Phys. Rev. Lett..

[18] Linskens AF (1996). Two-photon Rabi oscillations. Phys. Rev. A.

[19] Magnier S, Persico M, Rahman N (1999). Rabi oscillations between dissociative molecular states. Phys. Rev. Lett..

[20] Hu CX (2021). Angle-resolved Rabi flopping in strong-field dissociation of molecules. Phys. Rev. A.

[21] Gupta AK, Neuhauser D (2001). Rabi-oscillations-induced multiharmonic emission in a Maxwell-Schrödinger study of a dense sample of molecules. Int. J. Quantum Chem..

[22] Bucksbaum PH (1990). Softening of the H_2_^+^ molecular bond in intense laser fields. Phys. Rev. Lett..

[23] Ergler T (2005). Time-resolved imaging and manipulation of H_2_ fragmentation in intense laser fields. Phys. Rev. Lett..

[24] McKenna J (2008). Enhancing high-order above-threshold dissociation of H_2_^+^ beams with few-cycle laser pulses. Phys. Rev. Lett..

[25] Staudte A (2009). Angular tunneling ionization probability of fixed-in-space H_2_ molecules in intense laser pulses. Phys. Rev. Lett..

[26] McKenna J (2009). Suppressed dissociation of H_2_^+^ vibrational states by reduced dipole coupling. Phys. Rev. Lett..

[27] Wu J (2013). Understanding the role of phase in chemical bond breaking with coincidence angular streaking. Nat. Commun..

[28] Xu H (2016). Coherent control of the dissociation probability of H_2_^+^ in ω-3ω two-color field. Phys. Rev. A.

[29] Hu HT (2016). Wavelength and intensity effects on the dissociation of H_2_^+^ in intense laser fields. Phys. Rev. A.

[30] Ji QY (2019). Timing dissociative ionization of H_2_ using a polarization-skewed femtosecond laser pulse. Phys. Rev. Lett..

[31] Kangaparambil S (2020). Generalized phase sensitivity of directional bond breaking in the laser-molecule interaction. Phys. Rev. Lett..

[32] Mi YH (2020). Clocking enhanced ionization of hydrogen molecules with rotational wave packets. Phys. Rev. Lett..

[33] He CX (2020). Laser-wavelength and intensity dependence of electron-nuclear energy sharing in dissociative ionization of H_2_. Phys. Rev. A.

[34] Liang H, Peng LY (2020). Quantitative theory for electron-nuclear energy sharing in molecular ionization. Phys. Rev. A.

[35] Cao PK (2022). Resolving the time evolution of the dissociative nuclear wave packet in the repulsive state of H_2_^+^ via wave-packet interference. Phys. Rev. A.

[36] Sändig K, Figger H, Hänsch TW (2000). Dissociation dynamics of H_2_^+^ in intense laser fields: investigation of photofragments from single vibrational levels. Phys. Rev. Lett..

[37] Wu J (2013). Electron-nuclear energy sharing in above-threshold multiphoton dissociative ionization of H_2_. Phys. Rev. Lett..

[38] Lu PF (2017). Electron-nuclear correlation in above-threshold double ionization of molecules. Phys. Rev. A.

[39] Scully, M. O. & Zubairy, M. S. *Quantum Optics* 151–154 (Cambridge University Press, Cambridge, 1997).

[40] Pan SZ (2022). Low-energy protons in strong-field dissociation of H_2_^+^ via dipole-transitions at large bond lengths. Ultrafast Sci..

[41] Chu SI (1981). Floquet theory and complex quasivibrational energy formalism for intense field molecular photodissociation. J. Chem. Phys..

[42] Chu SI, Telnov DA (2004). Beyond the Floquet theorem: generalized Floquet formalisms and quasienergy methods for atomic and molecular multiphoton processes in intense laser fields. Phys. Rep..

[43] Giusti-Suzor A (1990). Above-threshold dissociation of H_2_^+^ in intense laser fields. Phys. Rev. Lett..

[44] Zavriyev A (1990). Ionization and dissociation of H_2_ in intense laser fields at 1.064 *μ*m, 532 nm, and 355 nm. Phys. Rev. A.

[45] Jolicard G, Atabek O (1992). Above-threshold-dissociation dynamics of H_2_^+^ with short intense laser pulses. Phys. Rev. A.

[46] Frasinski LJ (2001). Counterintuitive alignment of H_2_^+^ in intense femtosecond laser fields. Phys. Rev. Lett..

[47] Giusti-Suzor A, Mies FH (1992). Vibrational trapping and suppression of dissociation in intense laser fields. Phys. Rev. Lett..

[48] Frasinski LJ (1999). Manipulation of bond hardening in H_2_^+^ by chirping of intense femtosecond laser pulses. Phys. Rev. Lett..

[49] Charron E, Giusti-Suzor A, Mies FH (1995). Coherent control of photodissociation in intense laser fields. J. Chem. Phys..

[50] Kosloff R, Tal-Ezer H (1986). A direct relaxation method for calculating eigenfunctions and eigenvalues of the Schrödinger equation on a grid. Chem. Phys. Lett..

[51] Press, W. H. et al. *Numerical Recipes: The Art of Scientific Computing*. 3rd edn, 1048–1049 (Cambridge University Press, 2007).

[52] Feuerstein B, Thumm U (2003). Fragmentation of H_2_^+^ in strong 800-nm laser pulses: initial-vibrational-state dependence. Phys. Rev. A.

[53] Dörner R (2000). Cold target recoil ion momentum spectroscopy: a ‘momentum microscope’ to view atomic collision dynamics. Phys. Rep..

[54] Ullrich J (2003). Recoil-ion and electron momentum spectroscopy: reaction-microscopes. Rep. Prog. Phys..

